# Early Head Specification in Xenopus laevis

**DOI:** 10.1100/tsw.2003.54

**Published:** 2003-08-02

**Authors:** Blue B. Lake, Kenneth R. Kao

**Affiliations:** Terry Fox Cancer Research Labs, Faculty of Medicine, Memorial University of Newfoundland, St. John's, NFLD., A1B 3V6, Canada

**Keywords:** head development, embryonic neuraxes, organizer, Xenopus, review, Wnt signaling

## Abstract

The head represents the most dorsal and anterior extent of the body axis. In *Xenopus*, the progressive determination of the head is an extremely complex process involving the activation and localized antagonism of a number of interdependent intracellular signaling pathways including the Wingless/Int-1 (Wnt), bone morphogenetic protein (BMP), and nodal-related pathways. The sequence of events that specify the head are: dorsal-ventral polarization and head organizer specification in the blastula; gastrulation; neural induction; and patterning of the anterior-posterior and dorsal-ventral neuraxes. Wnt signaling is required for the specification of the dorsal side initially but is then inhibited within the organizer once it has formed. Similarly, Wnt signaling is required along the length of the neural tube, but must be suppressed at its rostral end for normal brain development. Nodal signaling is also necessary for induction of the mesendoderm, but is subsequently suppressed in its dorsal-anterior extreme to specify head organizer. BMP signaling is required for ventral mesoderm and non-neural ectoderm, and must also be suppressed in the head organizer region and for the differentiation of the ventral midline of the neural tube. Thus, development of the head, and indeed the body plan in general, requires precisely timed and spatially restricted activation and repression of these signaling pathways.

## AXIS SPECIFICATION AND ORGANIZER FORMATION

### Establishment of the Dorsal-Ventral Body Axis

The head represents the most anterior and dorsal part of the body plan. In *Xenopus*, development of the body axes is dependent on the formation of Spemann's Organizer, which is set up by the intersection of two processes before gastrulation: dorsal-ventral polarization and mesendoderm induction[73,95,145,150]. Establishment of dorso-ventral polarity occurs during the first cell cycle following fertilization, by the corticocytoplasmic rotation[51,61]. The rotation causes the displacement of vegetal pole cytoplasm to the dorsal vegetal side of the embryo, which activates intracellular Wingless/Int-1 (Wnt) signaling and subsequent translocation of β-catenin in the dorsal vegetal region[24,26,82,131,143]. Thus, an early dorsal-to-ventral gradient of stabilized β-catenin is generated which specifies the dorsal-ventral axis of the embryo. Stabilized β-catenin molecules enter the nucleus and bind a transcriptional complex containing the co-regulator XTcf-3 associated with promoter elements of the target genes[70]. At the onset of zygotic transcription at the mid-blastula transition (MBT), these complexes activate transcription of dorsal-specific genes such as the homeobox genes *siamois* and *twin*, both associated with the vegetal organizing Nieuwkoop Center, which in turn directly activate genes specific to both head- and trunk-inducing regions in the overlying marginal zone.

The mesendoderm is induced to form by a signal that emanates from the vegetal pole, which induces endoderm in vegetal cells and mesoderm in the overlying marginal zone cells[103]. This signal is initiated by the maternal T-box transcription factor, VegT[210], which activates several TGFβ superfamily members, including the *Xenopus* nodal-related (Xnr) proteins, and Derrière[105,205]. Transduction of the Xnr-mediated signal to the nucleus involves pathways typical of SMAD-dependentTGFβ superfamily signaling[80,199]. In *Xenopus*, there is a proportional correlation between the strength of the SMAD signal with the concentration of stimulating ligand and consequent dorsal character of the induced tissue[16,66,67]. Thus, a higher concentration of ligand is associated with activation of dorsal-type genes. In the embryo, this requirement is met by the interaction of β-catenin with Xnr signaling which regulates the onset and possibly stability or intensity of the induction signal on the dorsal side[75,127,204]. Further, recent evidence demonstrates that the initiation of zygotic Xenopus nodal expression is dependent upon early β-catenin/T-cell factor (TCF)-dependent transcription occurring prior to the MBT[208]. The interaction of SMAD-dependent TGFβ signaling with Wnt signaling illustrates the existence of functional cross-talk to establish the Spemann Organizer and subsequently the dorso-ventral axis of the embryo.

## Early Specification of the Organizer

Early studies on the inductive properties of the organizer revealed that it is a nonhomogeneous tissue with distinct head- and trunk-inducing regions (reviewed in [73]). Spemann first demonstrated this using heterotropic ansplantation of dorsal blastopore lips into the blastocoeles of host embryos, which leads to the formation of conjoined twins[182]. The supernumerary axis had differing anterior to posterior character depending on the time after the start of involution, with young lips inducing heads and older lips inducing tails (reviewed in [69]). In 1952, Nieuwkoop[148] proposed that differing head- or trunk-inducing regions within the organizer existed based on different anterior/posterior distributions of a posteriorizing agent (transformer) that altered the positional identity of neuroectoderm established by a general anterior neural inducer (activator).

Regionalization of the organizer has been detected as early as late blastula stages with the establishment of the head-inducing region in a position vegetal to the trunk-inducing region by the onset of gastrulation[212, Figure 1]. This regionalization has its earliest basis in the differential activity of both mesendoderm-inducing TGF-β superfamily signals and the β-catenin effector Siamois. Siamois, expressed in dorsal vegetal cells[119] and to a lesser extent in marginal and animal cells[43], acts within a short temporal window during early blastula stages to

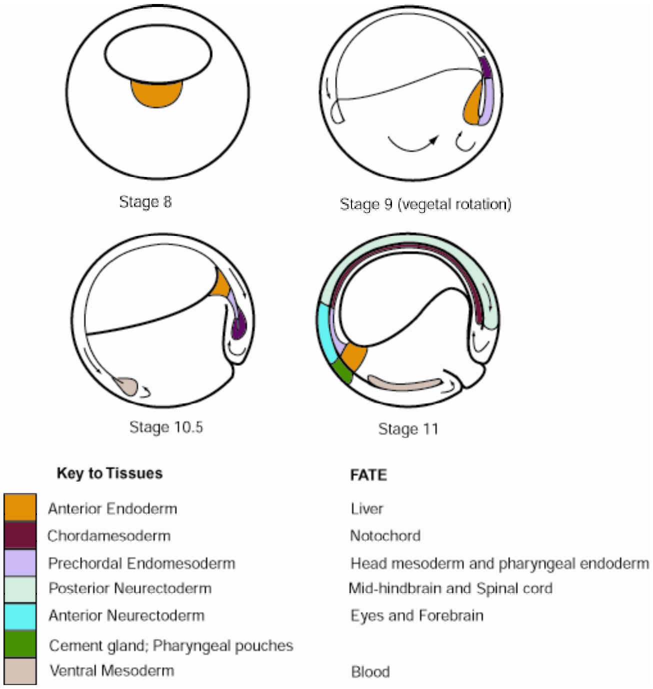

**FIGURE 1.** Differentiation of head organizer requires pregastrula interaction of the presumptive organizer with anterior endoderm. This figure illustrates the displacement of prospective anterior endoderm from deep within the vegetal half core to a position adjacent to the presumptive organizer by the process of vegetal rotation. It also shows the relative positions of anterior endoderm, prechordal mesoderm and chordamesoderm and their movement during gastrulation along the dorsal midline.

induce spatial- and concentration-dependent expression of genes of both the head and trunk organizers[105]. Such heterogeneity subdivides the gastrula organizer into three main domains, two of which directly contribute to head formation: the anterior (deep) endoderm, fated to form liver and the prechordal endo-mesoderm, which gives rise to prechordal (head) mesoderm and pharyngeal endoderm. The third domain, the chordal mesoderm, is trunk inducing and fated to form notochord. These domains are now fairly well characterized both morphologically and molecularly, with genetic profiles that are indicative of the associated inductive properties as well as prospective self-differentiation phenotype (Figures 1 and 2).

## Formation of the Head Organizer Requires Signals that Originate from the Anterior Endoderm (AE)

The anterior endoderm (AE) is derived from deep vegetal cells localized to the floor of the blastocoel and is topologically equivalent to the mouse anterior visceral endoderm (AVE), the chick hypoblast, and zebrafish dorsal yolk syncytial layer, each with differing contributions to head formation [as reviewed in ref. 41]. The AE has been implicated in head induction after it was found that it expresses the potent head inducer, Cerberus[17], as well as the fact that the AVE appears to be required for head induction in mouse. However, neither heterotopic transplantation nor ablation of the AE following the onset of gastrulation has demonstrated that it has distinct head-inducing activity[17,20,168], nor was it capable of neuralizing naïve ectoderm[17,58]. Quite possibly, the prospective AE functions prior to gastrulation, at a time when surgical manipulation is difficult. Consistent with this is the expression in the region, before

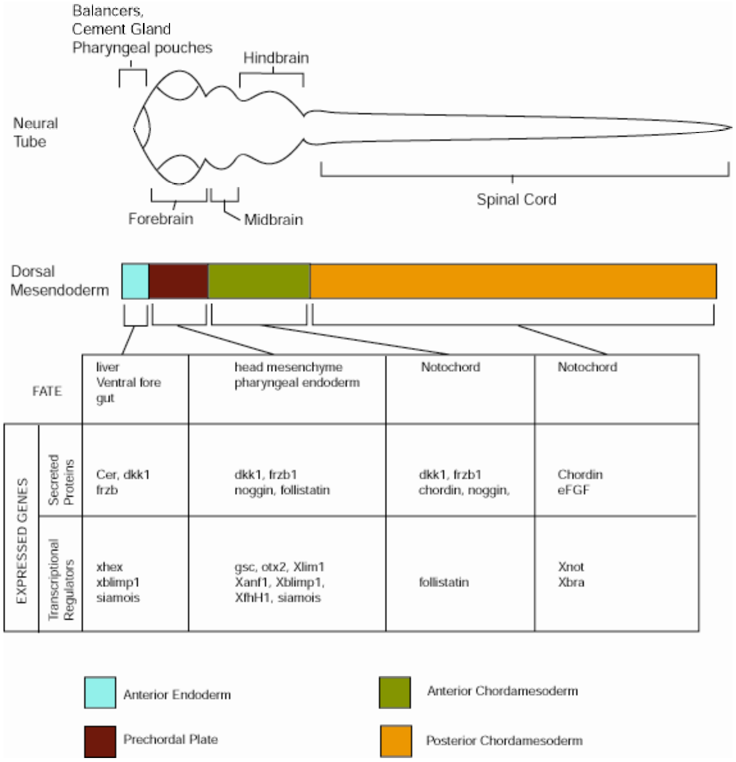

**FIGURE 2.** Expression patterns of secreted ligands and transcriptional regulators in the dorsal mesendoderm that specify anterior-posterior development in the nervous system of *Xenopus.*

gastrulation, of genes encoding inhibitors that are implicated in anterior specification, such as Cerberus (Cer), Hex, Dickkopf (Dkk1), and Frizbee-1 (Frzb-1) (Figure 2, Table 1).

How, then, does the AE influence head development? Following their establishment in centrally located deep endoderm, the Cerberus and Hex expressing prospective AE cells move from the central blastocoel floor to the dorsal side[92] via a pre-gastrula morphogenetic event, "vegetal rotation," a rearrangement of the vegetal cell mass that leads to expansion of the blastocoel floor and movement of the prospective AE in close apposition to the Organizer[201], where it becomes the AE proper (Figure 1). BMP antagonists from the organizer (noggin and chordin, Table 1) maintain Cerberus and Hex expression in the AE to perpetuate this anterior signal[214].

Early nodal-related signaling is required for the establishment of head organizing genes within the prechordal endomesoderm, but subsequently must be antagonized by inhibitors, such as Cerberus, to enable head formation[117,155]. Thus, the translocated anterior endoderm, through secretion of Cerberus, may titrate nodal signals in prospective head organizer (prechordal endomesoderm), which in turn provides BMP inhibitors to maintain the AE. This is likely the first complementary antagonistic interaction in the generation of distinct head and trunk organizers[53].

In *Xenopus*, the AE may be necessary, therefore, not directly for anterior neural induction, but for establishment of the head organizer through inhibition of trunk-inducing activity. Hex, for instance, is expressed in the AE and can achieve this function both nonautonomously through its ability to induce the expression of *Cerberus*[92,214] and cell autonomously by repressing genes necessary for the specification of trunk organizer, such as *goosecoid* and *chordin*[22]. A similar repression activity was described for the zinc finger transcriptional regulator, Xblimp1[40].

**TABLE 1**
**Antagonistic Partners in Embryonic Patterning**

| Extracellular Antagonist | Target(s) | Reference(s) |
|---|---|---|
| Cerberus | BMP4, Xwnt8, Xnr1 | [155] |
| Frzb-1 | Xwnt8, Xwnt1 | [120, 190, 191] |
| Dickkopf-1 | Xwnt3a, Xwnt8 | [63, 97] |
| WIF-1 | Xwnt8 | [86] |
| Noggin | BMP4 | [211] |
| Follistatin | BMP2, BMP4, BMP7 | [52, 87] |
| Chordin | BMP4 | [156] |

## The Prechordal Endomesoderm as Head Organizer

Nestled between the anterior endoderm and chordamesoderm is the prechordal endomesoderm, a potent head-inducing region (reviewed in [100]). During gastrulation, the prechordal endomesoderm moves anteriorly beneath the prospective neurectoderm until it lies under the anterior neural plate and forms the prechordal plate (Figure 1). Heterotypic grafting experiments in 1933 by Hilde Mangold (as cited in[147]) and later ablation studies[168] indicated that the prechordal plate is absolutely required for inducing forebrain and eyes in the overlying neural plate. Mediating this role are several potent head inducers expressed in the prechordal plate (Figure 2) such as Dkk1 and Frzb1[63,120,190]. While the chordamesoderm is primarily a trunk organizer, its signals are also required to specify caudal brain structures. Anterior chordamesoderm, as part of the head organizer, specifies the mid-hindbrain while posterior chordamesoderm, as part of the trunk organizer, specifies spinal cord[147].

## Maintenance of Nonhomogeneous Organizer

The maintenance of distinct head- and trunk-inducing centres requires a balance of Xnr, Wnt, and BMP signals and their associated antagonists. As mentioned above, both Wnt and Xnr signaling are required early to cooperatively induce the head and trunk organizers, but subsequently, in conjunction with BMP signals, their antagonism by anterior endoderm is necessary to delimit the head organizer. This antagonism distinguishes head- and trunk-inducing regions and maintains head organizer identity. In fact, simultaneous BMP and Wnt inhibition is sufficient to convert mesoderm into prechordal plate while the coordinated inhibition of nodal-related BMP and Wnt proteins is required for complete organization of the head as shown by the trivalent inhibitor Cerberus [63,64,155].

Several Wnt antagonists are expressed within the head organizer itself (Figure 2). These are necessary for formation of head structures by inhibition of post-MBT ligand-dependent Wnt signaling, the second phase of Wnt signaling in the embryo (subsequent to Wnt signaling that establishes the Nieuwkoop center) which antagonizes organizer mesendoderm and posteriorizes the neurectoderm (reviewed in [147]). Thus, when Xwnt8 was overexpressed in embryos after the MBT, the embryos lacked heads and notochords but had overrepresented somitic muscle[30].

Inhibition of Xwnt8 generated the opposite effect, whereby the embryos had enlarged heads and notochords at the expense of somitic muscle[84]. The highest concentration of Wnt antagonists lies within the head organizer where their expression originates, indicating that the absence of Wnt signals in this region defines the head organizer, while a lower level of antagonism in the chordamesoderm is required to specify trunk organizer. In fact, Dkk-1 expression in anterior mesendoderm was found to be both necessary and sufficient for specification of the head organizer[63,97]. Wnt antagonism, therefore, is vital not only for maintaining a balance between nonorganizer and organizer mesoderm, but also for maintaining the balance within the organizer between head- and trunk-inducing mesendoderm.

Recent evidence indicates that ventral BMP signaling, through activation of Xmsx1, suppresses the possibility of head structures developing ventrally by direct inhibition of nodal-related induction of Cerberus, Hex, and Dkk1[209], which are normally expressed in the anterior endoderm. BMP signaling also coordinately upregulates a pseudoreceptor, Bambi, which negatively regulates not only its own activity, but also that of nodal and activin by preventing formation of activated TGF$\beta$ superfamily receptor complexes[151]. Therefore, the ventral to dorsal gradient of BMP plays a role in maintaining the dorsal to ventral pattern of Xnr-induced tissues to define and spatially restrict the Organizer, while antagonism of BMP signals by Organizer-derived inhibitors (Figure 2, Table 1) is required to maintain both head and trunk structures.

Ventralizing BMPs, therefore, helps maintain the correct proportions of Organizer and non-Organizer mesoderm, while other BMP family members play a role in Organizer specification and differentiation. For example, the persistence of distinct, abutting, organizing centers involves a BMP family member, ADMP or anti-dorsalizing-morphogenic-protein. ADMP is expressed in chordamesoderm and antagonizes prechordal markers in that tissue possibly through repression of anti-Wnts[47]. Nodals, Wnts, and ADMP in the trunk organizer may inhibit head marker expression and promote trunk development while the head organizer secretes anti-Wnts (Cerberus, Dkk-1, Frzb-1, WIF-1, sFRP-1,2) and anti-nodal (Cerberus) to inhibit trunk while promoting head development[47]. Therefore, the coordinated action of BMPs, WNTs, Nodals and their associated antagonists define distinct head and trunk organizer regions by the onset of mesendoderm involution.

## NEURAL INDUCTION

### Pregastrula Specification

The majority of the neural plate is induced by signals derived from the organizer, except for ventral midline cells, also called the notoplate, which express the winged helix transcriptional regulator, Xfd-12′[53]. Notoplate cells are specified in the dorsal noninvoluting marginal zone prior to induction of the prospective neural plate, possibly by nodal-related ligands during mesendoderm induction. During pregastrula morphogenesis, at the same time during which the anterior endoderm is moved to the dorsal side by vegetal rotation, the animal hemisphere thins and stretches by epiboly, driving cells vegetally and translocating the future notoplate cells to the organizer in a domain abutting the prospective notochord[53]. These notoplate and notochord precursors are marked by their expression of Xfd-12′ and Xbra, respectively, both of which are dependent on FGF signaling[53,89,115,180]. These tissues are also required for convergent extension movements during gastrulation that are regulated by Wnt signaling[53,89,169,184]. At the onset of gastrulation, prospective notoplate cells undergo extensive mediolateral intercalation to bisect the basal neural plate anlage and position themselves along its ventral midline[99].

Induction of the remaining neural plate primordium occurs by vertical organizer-derived signals as the organizer migrates anteriorly beneath the prospective neural plate during gastrulation. These signals neuralize the ectoderm through inhibition of the default epidermal fate by antagonizing extracellular BMP (reviewed in [195]). However, ablation of the vertebrate organizer does not result in complete extinction of the neural plate[72], consistent with the observation that additional nonvertical processes acting through the plane continuous with the organizer and neural plate are involved in neural specification and patterning at a time prior to gastrulation[46]. Because dorsal ectoderm is already specified to form anterior neuroectoderm by late blastula stages, before the organizer has completely formed or undergone morphogenesis[44,68,124,173] it is possible that the planar signal acts through pregastrula suppression of BMP signaling in dorsal animal cap cells.

Recent studies have found that pregastrula asymmetry in the ectoderm results from dorsally stabilized β-catenin established following fertilization. Firstly, ectopic Wnt signaling represses transcription of BMP4 and concomitantly induces neural-specific markers in *Xenopus* ectoderm via a mechanism occurring outside the normal induction of the Wnt-responsive neuralizers, Xnr3 and Siamois[7]. Thus, these pre-MBT Wnt signals actually repress BMP mRNA expression to establish a domain of prospective neuroectoderm in the dorsal animal hemisphere, possibly acting to increase sensitivity to neuralizing signals from the organizer. Secondly, dorsally localized β-catenin activates early (blastula) expression of extracellular BMP antagonists such as chordin, noggin, follistatin, and Xnr3 in a "pre-organizer" region spanning the future organizer and possibly prospective neuroectoderm, independently of mesendoderm induction[196]. This activation is most likely mediated by the early activity of Siamois[104]. Thirdly, cleavage stage Wnt signaling establishes asymmetry in expression of the antineural homeodomain protein Dlx3, which becomes localized to ventral ectoderm while being repressed dorsally by early β-catenin[12]. The exclusion of this antineural factor from prospective neuroectoderm further adds to the proneural bias of pregastrula β-catenin activity, and strengthens the idea that the dorsal ectoderm is predisposed by the corticocytoplasmic rotation.

Therefore, the extracellular inactivation of BMP through planar signaling from the pre-organizer, in conjunction with β-catenin–dependent intracellular depletion of BMP and Dlx3 transcripts, represents the earliest events in neural plate formation. Subsequently, nodal-related signaling required for generation of the mesendoderm maintains expression of the BMP antagonists in the mature organizer. Their vertical secretion from mesendoderm to overlying prospective neurectoderm during gastrulation is required for maintenance and proper patterning of the prespecified neural plate. These studies underscore the importance of the combined action of planar and vertical signaling from the organizer to generate a fully patterned nervous system[21].

## Neuralization

By the onset of gastrulation, limited anterior-posterior (AP) character is bestowed upon the prospective neuroectoderm[58]. However, the extensive complexity of the vertebrate nervous system is stabilized and organized by signals emanating from underlying mesendoderm as it migrates and extends along the AP axis during gastrulation. The nonhomeogeneous nature of the organizer imparts positional character onto cells of the neural plate by establishing longitudinal and lateral domains of gene expression, which generate forebrain, midbrain, hindbrain, and spinal cord[58] (Figure 2).

Contraction of dorsal bottle cells that initiate upper blastopore lip formation triggers extensive morphogenetic rearrangement of tissues derived from each of the germ layers. Anterior endoderm is driven ahead of these involuting cells (Figure 1). In chick, this activity directs cell movements in the overlying ectodermal cells of the anterior-most neural plate that give rise to forebrain[55], physically segregating them from the caudalizing influence of the organizer[56]. However, post-blastula ablation of *Xenopus* anterior endoderm affected heart but not head formation[168], indicating a possible absence of further involvement of this tissue after organizer formation.

The AE may function alternatively in conjunction with the superficial epithelial cells (SBE-suprablastoporal endoderm) directly overlying the dorsal blastoporal lip[181], which become internalized during gastrulation to form the anterior archenteron roof and subsequent epithelial lining of the gut[98,176]. The SBE cells were found to transiently express Xhex at the onset of gastrulation[92], and targeted morpholino knockout of Xhex in animal cells generated severe anterior deletions[181]. Since the SBE is the earliest group of cells to involute and co-migrate with the AE beneath the ectoderm during gastrulation, it may function in combination to impart or maintain anterior identity that has been induced or physically segregated from more posterior tissues by the AE. A similar function was proposed for the corresponding mouse tissue, the anterior definitive endoderm (ADE), whose maintenance properties were believed to compliment the inducing properties of the AVE[2,13,137,174]. This cooperative function of these two tissues may explain why extirpation studies failed to show any dependence of the AE on head formation.

Following the AE and SBE, the cells of the presumptive prechordal plate exhibit spreading behavior on the ceiling of the blastocoel to occupy a position beneath the future forebrain (Figure 1). Further posteriorly, the chordamesoderm undergoes extensive medio-lateral intercalation which extends the body plan along the AP axis and positions its anterior end beneath the mid-hindbrain and its posterior end beneath the spinal cord (Figure 1).

Neural induction rResults from inhibition of BMP signaling mediated via BMPs -2,-4,-7, and GDF6 within the prospective neurectoderm[35,77,78]. The organizer provides the inhibitory signal by secreting BMP antagonists (Table 1) as it migrates anteriorly. Thus neural induction can be considered a developmental switch of ectoderm from an epidermal fate to the default neural fate resulting from active suppression of BMP signaling (reviewed in [195]) and concomitant alteration of the gene expression profiles as shown using microarray analysis[144]. The organizer-mediated extracellular sequestration of BMPs is stronger along the ventral midline, creating a slight morphogen gradient with the lowest levels of BMP signaling medially, higher levels at the lateral most edges of the neural plate and the highest levels in flanking epidermis. Intermediate BMP signaling is required to specify tissues found at the epidermal-neural border such as the cement gland[200] and neural crest[129].

Inhibition of BMP signaling may not be the only requirement for neural induction. The existence of a constitutive low strength FGF signal in ectodermal cells suggests that it is required for anterior neural induction following BMP depletion. This precondition would otherwise be either overcome by high levels of BMP signal for an epidermal fate or act in concert with lower BMP levels for cement gland fate[83].

## Establishment of Anterior-Posterior Pattern

The induction of neuroectoderm is intertwined with its AP patterning such that by late gastrula a neural plate is determined with a defined AP axis. This process involves a set of events that originates within the late blastula and continues through gastrula stages to progressively define and redefine gene expression patterns that provide positional identity. The dorsal ectoderm at the blastula stage is already specified as an anterior domain, resulting from planar signaling from the "pre-organizer region"[58]. Once gastrulation is initiated, two domains of AP character are soon established within the presumptive neuroectoderm: an anterior domain expressing the pan-neural marker opl and the anterior-specific marker otx-2 (forebrain); and a domain that is additionally expressing the more posterior marker fkh5, but not the hindbrain or spinal cord marker hoxD1[58]. At this stage, however, no stable neural determination has occurred, with presumptive neuroectoderm still capable of forming epidermis[91,94,179].

By mid-gastrula, the prospective neural plate has become determined[165,178,179] and exhibits a more elaborate AP pattern of three expression domains: an anterior domain (opl, otx2), a middle domain (opl, fkh5), and a posterior domain (opl, fkh5, hoxD1)[58]. Additional positional markers expressed at these time-points further define these domains[57] and demonstrate the sequential process of AP patterning that occurs concomitantly with neuralization. Differential exposure to quantitatively and qualitatively different secretory molecules derived from the underlying nonhomogeneous organizer induces distinct spatial expression patterns of these positional markers while at the same time cumulatively inducing general pan-neural markers[58].

## Wnt Antagonism Imparts Anterior-Posterior Polarity to the Neuraxis

The transforming signals that provide AP polarity to the neuraxis primarily involve Wnt antagonists (Cer, Dkk-1, Frzb-1, sFRP-1, 2, WIF-1), which are expressed at high levels in the prechordal plate and anterior chordamesoderm (Figure 2). Differential exposure to these anterior signals during gastrulation and upon final positioning under the neural plate generates a gradient of posteriorizing Wnt/β-catenin signals in the overlying neuroectoderm[101]. By late gastrula the Wnt gradient specifies a rudimentary AP pattern within the neuraxis that becomes refined, possibly through secondary cell–cell interactions, during neurula stages[101].

An absence or low level of Wnt signaling specifies the forebrain, while increasing levels of Wnts are required to specify increasingly posterior character. According to this model, the neural plate is innately fated to become anterior forebrain (telencephalon) and must be transformed by Wnt signals to posterior forebrain (diencephalon), midbrain, hindbrain, and spinal cord. This process likely involves the interplay of multiple Wnt ligands and their associated antagonists. For instance, paraxial mesoderm–derived Wnt8[11,30] and chordamesodermal Wnt3a[138] both exhibit long range signaling[101] and so may diffuse from these sources to generate a gradient within the gastrula neural plate. In addition, several Wnts are expressed more widely within the dorsal gastrula ectoderm, including Wnt3a[138], Wnt7b[27] and Wnt8b[33], which can act as the posteriorizing signal.

Depletion of the numerous Wnt ligands potentially present in both the head organizer and overlying neurectoderm requires antagonists of differing specificities. Dkk-1, which is both necessary and sufficient for promoting prechordal plate development, binds different Wnt ligands than Frzb-1[97]. This differential affinity enables Dkk-1 to specify the anterior-most endomesoderm, which subsequently patterns the ventral fore-midbrain[97]. It is for this reason that Dkk-1, unlike other Wnt antagonists, such as Frzb1, dnWnt8, and Cerberus, in conjunction with BMP inhibitors induces secondary heads with two eyes rather than one[17,63,64]. Once gastrulation has completed, the prechordal plate lies beneath the fore-midbrain where it specifies ventral fate. This is necessary for development of the ventral fore-midbrain cell types and cement gland, and the splitting of the eye field[121]. The importance of Dkk-1 in formation of ventral forebrain also supports the idea that Wnt signaling is involved not only in posteriorization but also dorsalization of the neural tube. Therefore, antagonism of Wnt signaling is required in the neuroectoderm to generate anterior ventral cell types, but must be preceded by proper specification of anterior endomesoderm endowed with the inductive properties of the head organizer.

While inhibition of canonical Wnt/β-catenin signaling is necessary for specification of both the anterior neural plate and underlying endomesoderm, proper migration and positioning of the prechordal plate or anterior midline cells is also required. This involves a balance between noncanonical Wnt signaling (β-catenin independent), possibly involving the Wnt 11 class of ligands (Wnts 4, 5a, 11) and their antagonists such as crescent[154], also known as Frzb-2[19]. In fact, overexpression of crescent, normally expressed in deep endoderm and prechordal endomesoderm, leads to a loss of ventral brain structures and fusion of the eyes due to disrupted anterior progression of axial mesendoderm during gastrulation[19,154]. Crescent appears to regulate non-β-catenin–dependent signaling by Wnts involved in cell morphogenetic events, implicating a role for Wnts and their antagonists not only in specification, but also migration of prechordal endomesoderm, both of which are necessary to establish a ventro-anterior brain phenotype.

## Molecular Characteristics of Wnt antagonists

The spatial restriction of the multiple Wnt ligands along the neuraxis involves antagonists derived from the anterior endoderm (Cerberus, Dkk1), prechordal endomesoderm/anterior chordamesoderm (Frzb-1, Dkk-1, sFRP-1, 2, WIF-1), and paraxial presomitic mesoderm (WIF-1) [17,63,86,120,190]. The three primary head inducers, Cerberus, Frzb-1, and Dkk1, each are structurally different, with overlapping yet distinct activities. Frzb-1 belongs to the frizzled-related protein (sFRP) family of secreted proteins that have a cysteine-rich domain (CRD), which is highly homologous to the Frizzled (Wnt-receptor) ligand binding domain[160]. This permits direct binding and sequestration of extracellular Wnt8 and Wnt1 proteins[120,190,191]. Similar binding also occurs with structurally dissimilar Cerberus and WIF1, both shown to directly bind Wnt8[86,155].

Dkk-1 is unique in that it shows a distinct mode of action through inhibitory interactions with a Wnt coreceptor, the LDL-receptor–related protein (LRP 5/6)[213]. Activation of the intracellular canonical Wnt/β-catenin is considered to involve Wnt binding to both the frizzled receptor and membrane bound LRP 5/6 to form a functional ligand-receptor-coreceptor complex[127,157,185,194]. Dkk proteins bind to the extracellular domain of LRP 5/6 with a higher affinity than frizzled and blocks formation of active trimeric Wnt-Frizzled-LRP5/6 complexes[6,128,170]. This unique mode of inhibition may account for the selective specificity of Dkk-1 for Wnt8 and Wnt3a[63,97], recapitulating the importance of the inhibitors having different specificities to antagonize cooperatively multiple Wnt ligands.

## Wnt Signaling Patterns the Brain

While Wnt signaling must be antagonized to allow proper specification of the forebrain and eyes, there is emerging evidence that components of this pathway are involved in its later patterning [102,153]. For instance, multiple constituents of the canonical Wnt pathway were found to be expressed within this region, including *XWnts-2B, -3A, and -8B* [33,113,202,203], *Xfzs-2, -3, -5, and -7*[38,175,183,197], *XTCF-3, LEF-1,* and *XTCF-4* [108,142]. Also, the Xfz3 receptor can promote ectopic eye formation, while its inhibition can suppress normal eye development[159].

Our recent work indicates that the novel Wnt pathway components Xenopus Pygopus (Xpygo)-2α and -2β, that likely mediate Wnt transcription through chromatin remodeling[14, 109,152,186], were found to be differentially expressed within the developing brain, with *Xpygo-2β* expressed within the retinal field and *Xpygo-2α* expressed throughout the prospective brain and eyes[112]. Antisense Morpholino (MO) knockdown experiments demonstrated that these differentially expressed *Xpygo-2* isoforms establish unique expression domains of both Wnt-responsive and Wnt-independent patterning genes required for correct A-P patterning of the fore-, mid-, and hindbrain[112]. Only a subset of Wnt-regulated markers were affected by antisense knockdown of these isoforms, indicating that not all Wnt-responsive markers require Xpygo-2 activity. Thus, differential expression along the neural tube of different Xpygo isoforms may provide an additional means of AP patterning by Wnt signaling in addition to variable local Wnt ligand concentrations.

## Non-Wnt–Dependent Anterior–Posterior Neural Patterning

Wnt signaling is not the only means of posteriorization, as BMPs, FGFs, and Retinoic acid (RA) have been implicated in this process[3,57,166]. Low doses of BMPs posteriorize neuroectoderm[34,93], but in a Wnt-dependent manner[97]. Aside from induction, at low levels, of anterior neural fate coincident with BMP antagonism, organizer derived FGF signaling subsequently imparts anterior-posterior positional identity to neural tissue through a posteriorizing mechanism that is intimately tied to the Wnt/β-catenin pathway[45,97,138].

Independent of Wnt signaling[97], RA provides positional identity primarily to hindbrain and spinal cord[15,107]. The availability of bioactive retinoids involves the cooperative action of two enzymes, RALDH-2 and CYP26. RALDH-2 is present in the posterior mesoderm of a gastrula embryo with a sharp border of expression at the level of the first anterior somite[28] and generates RA from retinaldehyde. On the other hand, CYP26, present in the gastrula prechordal mesoderm and overlying prospective anterior neural plate[39,81], breaks down RA and may promote its clearance[198]. Together these enzymes generate the appropriate boundaries of RA necessary to establish proper rhombomeric division of the hindbrain. The absence of RA in the anterior neural plate is required to promote appropriate development of this tissue through a mechanism of unliganded RAR-mediated repression of target genes[106].

## Establishment of Dorsal-Ventral Pattern

Establishment of the anterior-to-posterior (AP) and dorsal-to-ventral (DV) neuraxes are independent events, with AP patterning intertwined with neural induction during and preceding gastrulation to establish AP fate at open plate stages. Signaling for DV fate occurs later, after neural tube closure (reviewed in[3]). Signals from flanking, non-neural ectoderm, and underlying mesendoderm establish dorsal to ventral–oriented neuronal subtypes along the transversely segmented forebrain, midbrain, hindbrain and spinal cord. The epidermal signals, including BMP and Wnt ligands, initially induce lateral neural plate to give rise to neural crest and roof plate upon neural tube closure, the latter of which acts as a dorsal signaling center to generate a ventrally diffusing gradient of BMP signaling that, in conjunction with Wnt and FGF signals, specifies dorsal neuronal cell types[3,116,126].

The ventralizing signals derived from prechordal plate and notochord involve Sonic hedgehog (Shh) and nodal-related ligands that induce the ventral midline or floor plate. The floor plate subsequently generates a dorsally diffusing Shh gradient within the neural tube that acts as a spatial code for DV homeodomain gene expression needed to specify ventral neuronal progenitors[3,139,170]. Our recent evidence suggests that members of the Rel/NF-κB family of transcriptional regulators may be involved in ventral midline patterning. Ectopic expression of Xrel3 results in epidermal tumors that express ventral midline markers Shh and Gli1, and a dominant negative version of Xrel3 caused Shh- and Gli1-deficient embryos[111].

While the above paradigm is generally true for DV axial specification along the neural tube posterior to and including the midbrain, patterning of the forebrain seems to be more complex. While BMP signaling dorsalizes the posterior CNS, it also cooperates with Shh to induce ventral fate within the forebrain[36]. However, Shh retains a consistent role in specifying ventral identity, since a loss of Shh expression by a Rel/NF-κB inhibitor generates ventral midline abnormalities such as the inability to split the initially single eye field to form two bilateral eyes[111], consistent with studies in mouse[29].

Since the requirement for BMP signaling in the brain is restricted, its expression continues to be suppressed during neurula stages to establish discrete domains of activity that may further define expression boundaries of neural identity genes for specific populations of neuronal progenitor cells[74]. In support of this, several inhibitors of BMP transcription (BF-1, Xiro, Geminin) are expressed in defined domains of the anterior CNS[65,110,130]. These may restrict the suppressive activity of BMPs to defined domains which ultimately delineates the expression patterns of numerous neural markers. A similar process may also be involved for Wnt signaling, since gradients of Wnt receptors and antagonists are expressed in the developing mouse telencephalon[102].

The inductive properties of BMP in forebrain patterning indicate that additional factors for dorsalization must be involved. A novel secreted glycoprotein, Tiarin, has been identified that is expressed in the non-neural ectoderm surrounding the anterior neural plate and acts to dorsalize the neural tube independently of Shh, BMP, and Wnt signals[189]. Therefore, multiple signaling pathways collide to pattern the vertebrate CNS along both the AP and DV neuraxes.

## Secondary Brain Organizers

As described, early planar and vertical signals from the mesendoderm establish the basic AP pattern within the neurectoderm, defining broad territories of the forebrain, midbrain and hindbrain. However, as morphogenesis proceeds, the complex interconnected spatial and temporal neural proteome establishes transverse subdivisions of the forebrain into prosomeres and hindbrain into rhombomeres. In the forebrain, the superimposition on the established AP neuraxis of longitudinally aligned domains created by medial-lateral (ventral-dorsal) patterning generates a checkerboard or grid-like organization of the prosencephalic primordia [25,54,76,158,164]. Refinement and additional complexity of this fundamental pattern requires planar signals from three secondary organizing centers formed at the junctions of specified territories. Cooperative cellular interactions at these boundaries are believed to produce molecular signals with unique inductive capabilities[140].

The three main organizers present within the vertebrate central nervous system are: the anterior neural ridge (ANR), the zona limitans intrathalamica (ZLI), and the isthmic organizer (IsO). The ANR exists at the junction of the anterior neural plate and non-neural ectoderm[31,48], encompassing the first row of neural plate cells in zebrafish (row 1), and is required for anterolateral gene expression within the forebrain[85,172,177]. The ANR is characterized by its expression of FGF8 which initiates the expression of BF-1[177], a transcriptional regulator required for growth and regional specification of the telencephalic and optic vesicles[206]. Interestingly, Wnt signaling may also play a role in this process since XBF-1 expression was found to be dependent on Xpygo-2 within the anterior-neural plate[112].

While better characterized in mouse and zebrafish, the ANR recently has been found to play a conserved role in patterning and regionalizing the telencephalon and anterior-most diencephalon in *Xenopus*[49], a region, as in mouse, encompassing neurectoderm anterior to the ZLI[177]. The ZLI is positioned between dorsal (prosomere 2) and ventral (prosomere 3) thalamus[164]. This boundary was postulated to act as a morphogenetic barrier to segregate regions of different competence and prevent cell lineage mixing[114,132,136] while also acting as a morphogenetic source since it expresses the secreted protein Shh[10,50,158]. Anterior Shh expression in *Xenopus* was found to be dependent on the activity of a member of the Rel/NF-κB transcription regulator family which may localize Shh expression within the ZLI for a role in patterning neighboring forebrain tissues[111].

The most well-characterized secondary organizer exists at the isthmic constriction between the mes- and metencephalon (mid-hindbrain boundary or MHB), with a well-characterized function established in chick, mouse and zebrafish in patterning the midbrain and anterior hindbrain (cerebellum) from the diencephalon/midbrain boundary to and including the first rhombomere (reviewed in [4,133,162,193]). As such, transplantation experiments demonstrated the ability of the IsO to transform caudal prosencephalon to midbrain and rhombencephalon to cerebellum[8,9,60,90,132,135,136].

Several genes, across multiple species, have been identified as expressed in and required for the development of the IsO (reviewed in[162]) including those encoding transcriptional regulators (en1, en2, pax2, pax5, otx1, otx2, gbx2) and secreted proteins (Wnt1 and FGF8). Recent studies have also demonstrated the existence of similar genetic profiles with the associated interconnected dynamics and inductive capabilities in the *Xenopus* nervous system[62,163,187,188].

The position of the MHB boundary is established at the intersection of mutually repressive otx2 and gbx2 homeoprotein expression domains established during primary neural induction[59,79,88,96,134], which each independently are required for development of the fore-midbrain and hindbrain, respectively[1,2,5,192]. Both Xotx2 and Xgbx2 expression domains converge at the late gastrula stage enabling expression of XFGF8 within the overlapping region[62] which, in other species, is concomitant with or preceded by the independent expression of pax2 and Wnt1 in a co-localized transverse band[125,161]. Through positive feedback with Xgbx2 and negative feedback with Xotx2, XFGF8 propagates the formation of a sharp and exclusive Xotx2/Xgbx2 boundary[62] as in other vertebrate systems[59,79,122,123,134]. This sharp boundary was postulated to maintain FGF8 expression and enable its activation and maintenance of IsO genes, such as pax2, en1, en2, and Wnt1[32,59,122]. Overexpression of either Xotx2 or Xgbx2 generates a caudal or rostral shift, respectively, of the opposing marker's expression domain with the associated alteration of MHB-specific markers[62] reminiscent of studies in mouse and zebrafish[23,96,141]. Further, Xotx2 was found to be capable of independently inducing a large component of the MHB network with correct spatial positioning along the AP neuraxis dependent on mutual negative regulation with Xgbx2 occurring at mid- to late gastrula stages[187,188].

Establishment of the abutting Xotx2/Xgbx2 expression domains and resultant isthmic specification in *Xenopus* further requires the homeoprotein Xiro[62]. Xiro maintains Xotx2 expression in the prospective midbrain by reciprocal activation at the gastrula stage before additionally activating Xgbx2 expression at the late gastrula/early neurula stage in the future hindbrain[62]. Xiro is also necessary for XFGF8 induction at the isthmus and XFGF8-induced expression of Xen2 in the Xotx2 expressing territory[62]. en2 expression is required for growth and polarization of the mesencephalon for proper retinotectal map formation and is regulated by both FGF8[118,123] and Wnt1[37] through Xpygo-2$\alpha$ protein complexes in *Xenopus*[112]. Like FGF8, en2 but not Wnt1 has the ability to ectopically induce the IsO genetic complement[42,163], as required for a later maintenance phase whereby the reciprocal associations between en2, FGF8, pax2, and Wnt1 are required for their continued expression[122,123,161,171].

Once established at the proper position, the IsO not only patterns both the midbrain and anterior hindbrain, but also acts as a mitogenic source to control the size and shape of the adjacent tissues through secretion of Wnt1 from the midbrain/otx2 side and FGF8 from the hindbrain/gbx2 side (reviewed in[133]). Therefore, the complex interconnected genetic network required for the initiation and maintenance of the IsO induces the cellular diversity of mid- and anterior hindbrain for development and outgrowth of higher order brain structures: the retinotectal map and cerebellum.
